# A Benchmark Comparison of Four Off-the-Shelf Proprietary Visual–Inertial Odometry Systems

**DOI:** 10.3390/s22249873

**Published:** 2022-12-15

**Authors:** Pyojin Kim, Jungha Kim, Minkyeong Song, Yeoeun Lee, Moonkyeong Jung, Hyeong-Geun Kim

**Affiliations:** 1 Department of Mechanical Systems Engineering, Sookmyung Women’s University, Seoul 04310, Republic of Korea; 2 Department of Mechanical Engineering, Incheon National University, Incheon 22012, Republic of Korea

**Keywords:** visual navigation, visual–inertial odometry, Apple ARKit, Google ARCore, VIO

## Abstract

Commercial visual–inertial odometry (VIO) systems have been gaining attention as cost-effective, off-the-shelf, six-degree-of-freedom (6-DoF) ego-motion-tracking sensors for estimating accurate and consistent camera pose data, in addition to their ability to operate without external localization from motion capture or global positioning systems. It is unclear from existing results, however, which commercial VIO platforms are the most stable, consistent, and accurate in terms of state estimation for indoor and outdoor robotic applications. We assessed four popular proprietary VIO systems (Apple ARKit, Google ARCore, Intel RealSense T265, and Stereolabs ZED 2) through a series of both indoor and outdoor experiments in which we showed their positioning stability, consistency, and accuracy. After evaluating four popular VIO sensors in challenging real-world indoor and outdoor scenarios, Apple ARKit showed the most stable and high accuracy/consistency, and the relative pose error was a drift error of about 0.02 m per second. We present our complete results as a benchmark comparison for the research community.

## 1. Introduction

Visual–inertial odometry (VIO) is the process of determining the position and orientation of a camera and inertial measurement unit (IMU) rig in 3D space by analyzing the associated camera images and IMU data from the visual–inertial sensors. It is one of the fundamental building blocks for 6-DoF ego-motion estimation in a variety of domains [1,2,3,4], including autonomous vehicles and virtual and augmented reality (VR/AR). VIO approaches are popular choices for producing labeled training data of 6-DoF camera poses when developing neural inertial navigation algorithms [5,6,7] due to their ability to operate without a motion capture system or laser tracker. As the VIO research has reached a level of maturity, there exist several open and published VIO methods, such as MSCKF [8], OKVIS [9], and VINS-Mono [10], and many commercial products utilize closed and proprietary VIO algorithms, such as Apple ARKit [11] and Google ARCore [12], which offer off-the-shelf VIO pipelines that can be employed on an end-user’s system of choice. VIO sensors are essential for various experimental environments that require 6-DoF motion tracking, and many engineers and researchers have tried to find appropriate and feasible VIO sensors and algorithms for their robotic systems and applications.

Recent research has provided some comparative experiments on the performance of popular VIO approaches, but the authors only considered a subset of the existing open-source and proprietary VIO algorithms and conducted insufficient performance evaluations on only publicly available datasets, rather than challenging real-world indoor and outdoor environments. In particular, although commercial VIO sensors (Intel T265, Stereolabs ZED 2) have played an important role in several DARPA challenges [13,14] and many commercial products or apps (Pokémon GO, IKEA Place AR), there is a lack of research for the benchmarking of the positioning accuracy of these closed and proprietary VIO platforms.

**Figure 1:**
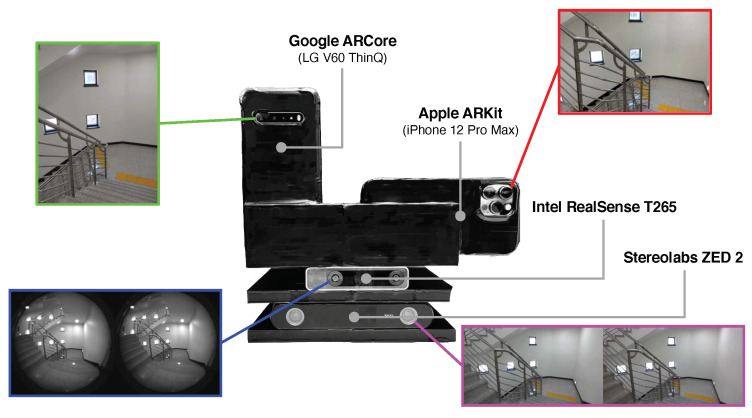
The custom-built rig for benchmarking the 6-DoF motion-tracking performance of four visual–inertial (VI) sensors: Apple ARKit (iPhone 12 Pro Max), Google ARCore (LG V60 ThinQ), Intel RealSense T265, and Stereolabs ZED 2.

The motivation of this paper is to address this deficiency by performing a comprehensive evaluation of off-the-shelf commercially available VIO sensors in challenging indoor and outdoor environments, as shown in Figure 1. This is the first comparative study on four popular proprietary VIO sensors in six challenging real-world scenarios, both indoors and outdoors. In particular, we selected the following four proprietary VIO systems, which are frequently used in 6-DoF motion-tracking problems:

- Apple ARKit [11]—Apple’s augmented reality (AR) platform, which includes filtering-based VIO algorithms [15] to enable iOS devices to sense how they move in 3D space.
- Google ARCore [12]—Google’s AR platform that utilizes a multi-state constraint Kalman filter (MSCKF)-style VIO algorithm [8,16], which is called concurrent odometry and mapping (COM) [17].
- Intel RealSense T265 [18]—a stand-alone VIO and simultaneous localization and mapping (SLAM) tracking device developed for use in robotics, drones, and more, with all position computations performed on the device.
- Stereolabs ZED 2 [19]—a handheld stereo camera with a built-in IMU for neural depth sensing and visual–inertial stereo; it requires an external NVIDIA GPU to obtain the 6-DoF camera poses.

Our key contribution is the qualitative and quantitative evaluation of four popular commercial VIO sensors in challenging real-world 6-DoF motion-tracking scenarios, which have not been performed in previous papers. We focus on commercial off-the-shelf VIO sensors that might be easy to use and of interest to more researchers and engineers because the open-source VIO methods that have been published [2,10,20,21] are relatively difficult to understand and operate, and comparisons thereof have been made in the literature [1,22,23,24] to some extent. We do believe that our scientific contributions will be of great help to researchers and engineers seeking appropriate and feasible VIO sensors for their robotic systems and applications.

**Figure 2:**
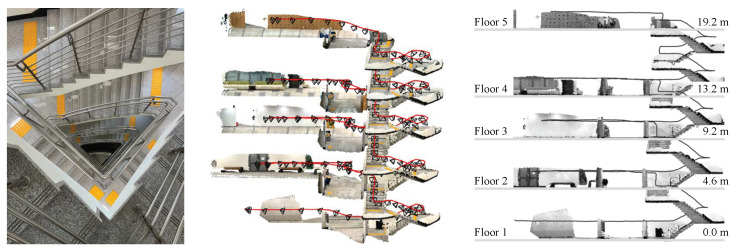
Accumulated 3D point cloud (**middle**) with the estimated 6-DoF trajectory (red) from Apple ARKit in multi-floor environments. We captured the 6-DoF camera poses and 3D points while climbing the multi-story stairs (**left**). Among the four proprietary VIO systems, Apple ARKit showed the most consistent and accurate 6-DoF motion-tracking results, as it consistently reconstructed the 3D geometry of stairs and hallways. The track (red) and 3D reconstruction results of Apple ARKit had a similar shape to that of the ground-truth blueprint of the building (**right**).

Our experiments were conducted in six challenging indoor and outdoor environments with a custom-built test rig equipped with the four VIO sensors, as illustrated in Figure 1 and Figure 2. Our test sequences contained long and narrow corridors, large open spaces, repetitive stairways, an underground parking lot with insufficient lighting, and about 3.1 km of a vehicular test in a complex urban traffic environment. In order to further increase the reliability and credibility of our experiments and conclusions, we performed additional comparative experiments with the ground-truth trajectories from OptiTrack motion capture systems.

## 2. Related Work

Despite proprietary VIO sensors being utilized in many products and areas for industrial usage (e.g., for building an accurate indoor map, as a precise positioning system, etc.), there is no benchmark study that satisfies our proposed goals. While comprehensive comparisons of open-source VIO methods that have been published exist [1], they focused only on the evaluation of popular academic VIO algorithms on the EuRoC micro aerial vehicle dataset [25] and did not cover off-the-shelf proprietary VIO sensors and various indoor and outdoor environments. Although ADVIO [26] presented a VIO comparison that included three proprietary platforms and two academic approaches, its main contribution was the development of a set of RGB and IMU smartphone datasets, not a performance evaluation among proprietary VIO sensors. In [27,28,29], some comparative studies of proprietary VIO sensors were performed, but they considered only a few proprietary VIO platforms and focused only on the evaluation of the performance of 2D planar camera movements in a simple indoor environment with no height changes. A performance evaluation was only conducted in a simple 2D indoor environment with a short camera moving distance.

Since we are focused on the 6-DoF positioning accuracy of proprietary VIO sensors, we can instead consider the existing results that are relevant to this problem. The VIO approach proposed in [30] was compared to Google ARCore and VINS-Mono [10], but only on a few indoor sequences with very little camera movement. In [24], ARCore, ARKit, and T265 were qualitatively compared with the proposed VIO method only on non-public and simple 2D planar datasets. The evaluation framework in [31] assessed the 6-DoF motion-tracking performance of ARCore with the ground truth under several circumstances, but they lacked comparative results for other proprietary VIO systems, such as ARKit and T265, and detailed analyses were performed only for ARCore.

Most important is that no existing work considered an indoor/outdoor performance evaluation for four popular proprietary VIO systems that are frequently deployed in robotic applications, AR/VR apps, and industrial usages. Our test sequences are authentic and illustrate realistic use cases, as they contain challenging environments that are both indoors and outdoors with scarce or repetitive visual features and varying motions, from walking to driving camera movements. They also include rapid rotations without translation, as these are problematic motions for many VIO/SLAM algorithms. Our work is the first to address this need.

## 3. Commercial Visual–Inertial Odometry Sensors

We briefly summarize the primary features of four off-the-shelf proprietary VIO sensors based on data published on the relevant official websites, papers, GitHub, and patent documents, as well as how the 6-DoF pose estimates are collected from each VIO mobile sensor. Since most proprietary VIO/SLAM platforms are closed-source, we do not cover the detailed academic backgrounds and implementations of VIO.

### 3.1. Apple ARKit

Apple ARKit [11] is Apple’s augmented reality (AR) software framework, and it includes a tightly coupled filtering-based VIO algorithm that is similar to the MSCKF [8] in order to enable iOS devices to sense how they move in 3D space. It contains a sliding-window filter, bundle adjustment, and motion/structure marginalization modules [15], and it is expected to be applied to various robotic applications, such as the Apple Glasses and Car, in the future, and is not just for the iPhone and iPad, which is why we conducted vehicle tests in this benchmark. We developed a custom iOS data collection app (https://github.com/PyojinKim/ios_logger, accessed on 19 October 2022) for capturing ARKit’s 6-DoF camera poses, RGB image sequences, and IMU measurements by using an iPhone 12 Pro Max running iOS 14.7.1. This saved the pose estimates as a translation vector and a unit quaternion at 60 Hz, and each pose was expressed in a global coordinate frame created by the phone when starting iOS data collection. Although there are various iPhone and iPad models, the core VIO algorithm in ARKit is the same; thus, we empirically confirmed that there is little difference in the VIO performance of each device.

### 3.2. Google ARCore

ARCore [12] is Google’s platform for building AR experiences by utilizing multi-state constraint Kalman filter (MSCKF)-style VIO/SLAM algorithms [16,32] with many subsequent variations, which is called concurrent odometry and mapping (COM) [17]. ARCore is a successor to Google Project Tango [33], and it is currently applied only in Android OS smartphones, but it will be extended to various robotic platforms, such as Google Wing, Maps, and Waymo, which is why we evaluated ARCore in a large-scale outdoor sequence of about 3.1 km in a vehicular test. We built a custom Android OS app based on Google’s ARCore example (https://github.com/rfbr/IMU_and_pose_Android_Recorder, accessed on 19 October 2022) to acquire ARCore’s 6-DoF camera poses and IMU measurements at 30 Hz with an LG V60 ThinQ running Android 10.0.0 and ARCore 1.29. Although there are various Android OS devices, such as the Samsung Galaxy and Google Pixel, smartphones on the list (https://developers.google.com/ar/devices, accessed on 19 October 2022) certified by Google demonstrate similar motion-tracking performance regardless of the device model.

### 3.3. Intel RealSense T265

Intel RealSense T265 is a hassle-free stand-alone VIO/SLAM device that tracks its own position and orientation in 3D space. The embedded processor, a vision processing unit (VPU), runs the entire VIO algorithm onboard, analyzes the image sequences from stereo fisheye cameras, and fuses all sensor information together. Since the T265 VIO algorithm runs on the device itself without using the resources of a host computer, it is widely used as a 6-DoF positioning sensor in 3D space for various robotic applications, such as DARPA challenges [13] and autonomous flying drones [34]. We collected the 6-DoF motion tracking results at 200 Hz by using Intel RealSense SDK 2.0 (https://github.com/IntelRealSense/librealsense, accessed on 19 October 2022) and saved the T265’s 6-DoF camera poses by connecting it to an Intel NUC mini-PC.

### 3.4. Stereolabs ZED 2

Stereolabs ZED 2 is a handheld stereo camera with a built-in IMU for neural depth sensing, 6-DoF VIO/SLAM, and real-time 3D mapping. Stereolabs has not made their VIO/SLAM algorithm public, and the description of the VIO algorithm is relatively vague compared to those of other proprietary VIO systems. It is a popular stereo camera sensor for various robotic applications, such as drone inspection [35], but it has the disadvantage of requiring an external NVIDIA GPU to perform positional tracking and neural depth sensing. We developed a program to collect the ZED 2 6-DoF camera poses at 30 Hz based on ZED SDK 3.5.2 (https://www.stereolabs.com/developers/release/, accessed on 19 October 2022) on an NVIDIA Jetson Nano onboard computer.

## 4. Experiments

**Figure 3:**
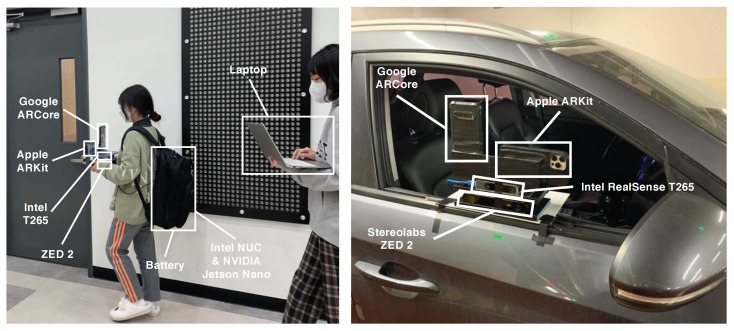
We carried the custom-built capture rig by hand and stored the computers and batteries to collect the indoor 6-DoF motion data from the VI sensors (**left**). In the outdoor vehicular tests, we fixed the capture rig to the front passenger seat (**right**).

**Figure 4:**
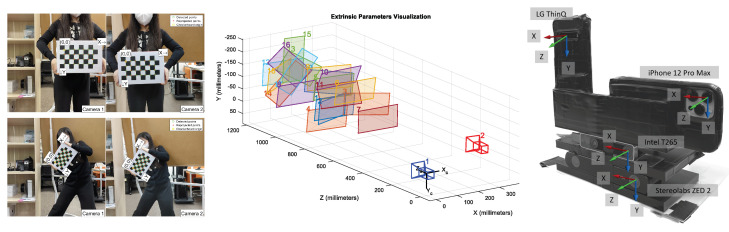
Intrinsic and extrinsic calibration of all cameras. We calibrated the intrinsic and extrinsic camera parameters of the four tested VIO sensors by capturing multiple views of a checkerboard. We utilized MATLAB’s built-in camera calibration toolbox and visualized the custom-built sensor rig with orientations of all four VIO sensors.

We both qualitatively and quantitatively evaluated the four proprietary VIO sensors with the four devices (iPhone 12 Pro Max, LG V60 ThinQ, Intel T265, ZED2) attached to a custom-built capture rig, as shown in Figure 3, in large-scale and challenging indoor and outdoor environments. Indoors, we recorded the motion data with a walking person, and outdoors, the data were collected by rigidly attaching the capture rig to a car, as shown in Figure 3. We saved the 6-DoF pose estimates of ARKit and ARCore through the custom apps on each smartphone device, and we recorded the moving trajectories of T265 and ZED2 in the Intel NUC and NVIDIA Jetson Nano onboard computers. We maintained the default parameter settings of each VIO sensor and deactivated all capabilities related to SLAM (e.g., loop closure) for a fair comparison of each VIO system. Furthermore, in order to interpret the motion-tracking results in the same reference coordinate frame, we calibrated the intrinsic and extrinsic camera parameters of all four VIO sensors by capturing multiple views of a checkerboard pattern [26,36], as shown in Figure 4. Given the checkerboard images that were taken with each VIO sensor, we obtained intrinsic and extrinsic calibration parameters by using MATLAB’s built-in camera calibration toolbox.

**Figure 5:**
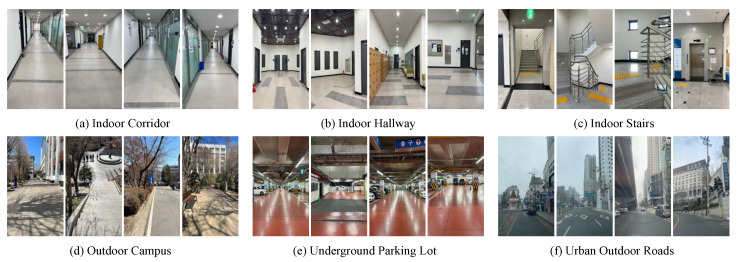
Example images from the indoor and outdoor benchmark datasets. The top row presents three indoor sequences that were traversed by foot, which included long corridors (**a**), open hallway spaces (**b**), and repetitive stairs (**c**) in a university building. We acquired the camera motion data on the outdoor campus on foot (**d**) and in a car in an underground parking lot (**e**) and on urban outdoor roads (**f**).

Our benchmark dataset contained various indoor and outdoor sequences in six different locations, and the total length of each sequence ranged from 83 to 3051 m; this was primarily designed for the benchmarking of medium- and long-range VIO performance. There were three indoor and three outdoor sequences, and all indoor sequences were captured in a seven-story building in the university campus; it included long corridors, open hallway spaces, and stair climbs, as shown in the top row of Figure 5. The indoor cases were as realistic as possible; they contained repetitive motion on stairs, temporary occlusions, and areas lacking visual features. The bottom row of Figure 5 illustrates example frames from three outdoor sequences that were acquired outdoors on the university campus, in underground parking lot, and on urban roads. In order to quantitatively evaluate the performance of each VIO system without an external motion capture system, we had the start and end points of the movement trajectories in all experiments coincide, and we measured the final drift error (FDE) metric, which was the end-point position error in meters. We report the quantitative evaluation results of the four VIO sensors in Table 1. The smallest end-point position error for each sequence is indicated in bold. The ideal FDE value (the ground-truth path) should be 0, and a large FDE value denotes an inaccurate position estimate, since we define the starting point of the movement as the origin. In addition, by overlaying the estimated VIO trajectories on the floor plan of the building or Google Maps, we qualitatively evaluated the consistency, stability, and reliability of each VIO system.

### 4.1. Long Indoor Corridors and Open Hallways

**Figure 6:**
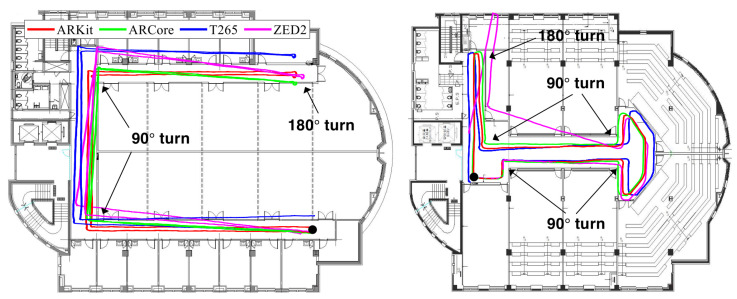
Trajectories estimated by the four proprietary VIO systems in the long U-shaped corridor (**left**) and open hallway space (**right**) sequences. We started and ended at the same point, which is marked with a black circle, to evaluate the loop-closing performance of the tested commercial VIO systems. The 90° and 180° turns gauged the ability of the VIO algorithms to handle rotations in the yaw direction. The estimated paths for ARKit (red) matched the building floor plan most consistently, and only the starting and ending points of ARKit nearly met; for the others, they did not.

We evaluated the four VIO sensors in a long U-shaped corridor and in open hallway spaces that are easily found in typical office and university buildings, as shown in Figure 6. Figure 5a,b illustrate example frames from both locations. The trajectories of these sequences were approximately 145 and 84 m, and they included 5 and 11 pure rotational movements and difficult textures. The left side of Figure 6 shows the trajectories of the 6-DoF motion-tracking results with the four VIO sensors with movements that went from end to end of a long U-shaped corridor and then returned back to the starting point. The 180° turn gauged the ability of VIO algorithms to handle rotations in the yaw direction. The right side of Figure 6 shows the trajectories of a large turn along the wall, which included many rotations in place in an open hallway space.

In the long U-shaped corridor and open hallway sequences, the start and end points of ARKit (red) met at the black circle without a severe rotational drift, while the orthogonality and scale of the estimated trajectory were well maintained in comparison with the floor plan. Although ARCore (green) showed the most accurate results in terms of the FDE metric, as shown in Table 1, the estimated VIO trajectory did not match the floor plan well. Intel T265 (blue) estimated accurate 3-DoF rotational motion well, but there was a problem with the scale of the moving trajectory in comparison with the floor plan, as it showed a slightly larger trajectory than the actual movements. ZED2 (magenta) presented the most inaccurate and inconsistent positioning performance among the four VIO methods, as the rotational motion drift error gradually accumulated over time. Overall, the VIO trajectories estimated by ARKit (red) were the most similar and consistent motion tracking results with respect to the actual movements that followed the shape of the corridor on the floor plan.

### 4.2. Indoor Multi-Story Stairs

**Figure 7:**
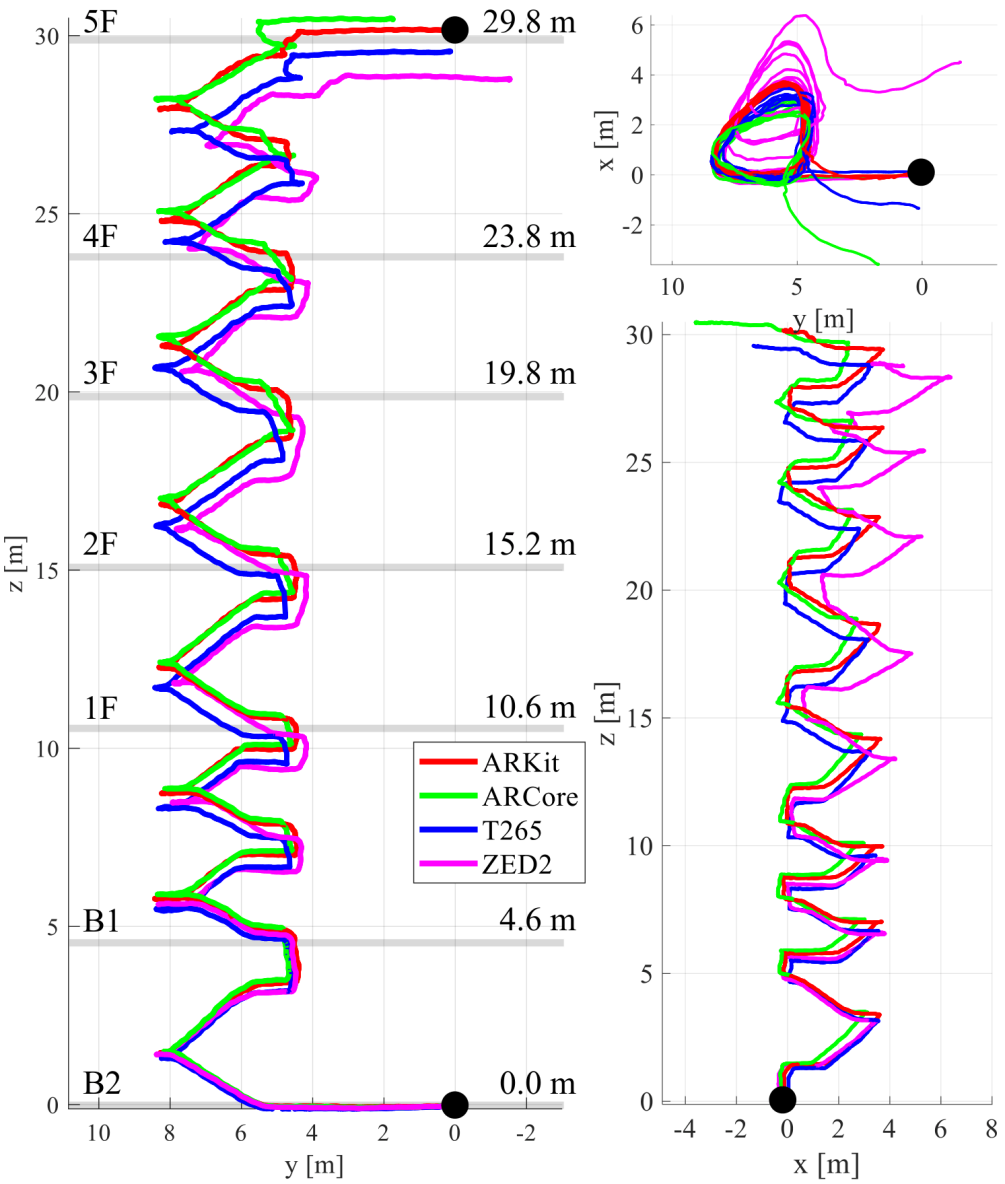
Comparison of the four VIO systems on multi-story stairs from the second basement floor (B2) to the fifth floor (5F). The side (**left**), front (**bottom right**), and top (**top right**) views of the estimated VIO trajectories are shown. ARKit had the camera motions that were most consistent with the shape of the stairs, and it only matched the start and end points, which are marked in the black circle.

We performed a comparative experiment in a multi-floor staircase environment with a 114 m trajectory going up the stairs from the second basement floor (B2) to the fifth floor (5F) of a building, as shown in Figure 7. The repetitive rotational motion included in the 3D trajectory of climbing the stairs made VIO positioning challenging. Figure 5c shows example frames from the multi-story stair sequence. In the top view (xy-plane), we started and ended at the same points marked in the black circle to check the loop closing in the estimated VIO trajectories. ARKit (red) had the best performance; the top and side views of ARKit (red) show the overlapped, consistent 6-DoF motion-tracking results, while other VIO systems gradually diverged from the initially estimated loop. With ARKit (red), the starting and ending points in the xy-plane (top view) nearly matched; for the others, they did not. The final drift error (FDE) of ARKit in the xy-plane was 0.19 m, while those of ARCore, T265, and ZED2 were 3.98, 1.49, and 4.76 m, respectively. In particular, ZED2 (magenta) had the most severe trajectory distortion in the z-axis direction (height) among the four VIO systems. Figure 7 illustrates the side and front views of the stairway with the paths from the four VIO devices, showing the high consistency of ARKit (red) compared to that of the other VIO platforms. It is noteworthy that the height of each floor estimated by ARKit and the actual height (the ground truth) from the building blueprint were approximately identical. Please refer to the Video S1 clips submitted with this paper showing more details about the experiments.

### 4.3. Outdoor University Campus

**Figure 8:**
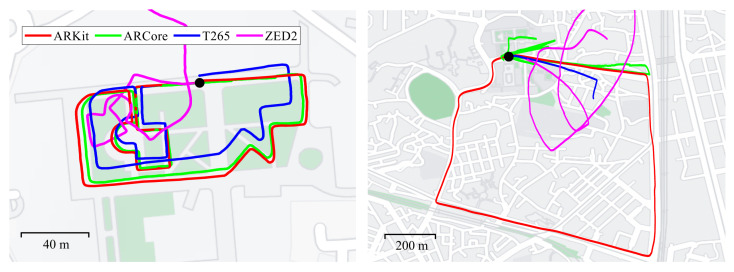
Estimated motion trajectories of four proprietary VIO systems in the outdoor campus (**left**) and urban outdoor roads (**right**) sequences overlaid on Google Maps. We started and ended at the same point, which is marked in the black circle, to check the loop-closing performance. ARKit (red) tracked the 6-DoF camera poses well, following the shape of the roads on Google Maps most consistently and accurately. Only ARKit (red) was able to produce stable motion-tracking performance even when driving a vehicle over 60 km/h (**right**).

We chose an outdoor location in the university campus with a length of approximately 513 m to determine which VIO system worked well in an environment with a rapid change in the topography, in addition to a narrow returning road, as shown on the left side of Figure 8. Example frames are shown in Figure 5d. The main purpose of choosing the university campus was to evaluate which VIO sensor worked well in the daily life environments around us, which are crowded with people and contain narrow roads and stairs. In addition, we intentionally matched the starting and ending points and designed the moving trajectories for the experiments by referring to Google Maps and the university campus map.

The resulting 6-DoF trajectories from four VIO platforms are shown overlaid on Google Maps, demonstrating that the start and end points of ARKit (red) and ARCore (green) met while matching well with the shape of the roads shown on Google Maps. The shape of the estimated trajectory of T265 (blue) was very similar to ARKit’s and ARcore’s results, but the scale of the estimated path of T265 was smaller than the actual movements. T265 suffered from a scale inconsistency problem, which is generally observed in monocular visual odometry configurations. The orthogonality of ZED2 (magenta) was broken due to its inaccurate rotation estimation, showing the most severe distortion of the actual movement trajectory among the four VIO systems, as shown on the left side of Figure 8.

### 4.4. Outdoor Urban Roads and Parking Lot

We performed an outdoor vehicle driving experiment with a mileage of approximately 3 km by attaching the capture rig to a vehicle, as shown on the right side of Figure 8. Figure 5e,f show example frames from the underground parking lot and urban outdoor roads. We acquired motion data while driving on public automobile roads near Seoul Station in Seoul, and there were plenty of moving people, cars, and, occasionally, large vehicles that were visible in the outdoor environments, which made motion tracking with VIO challenging. Even in high-speed driving conditions, sometimes exceeding 60 km/h, ARKit (red) showed surprisingly accurate and consistent 6-DoF motion-tracking results when overlaid on Google Maps, as shown on the right side of Figure 8. The start and end points of ARKit (red) accurately met in the black circle, and the final drift error (FDE) was only 2.68 m, as shown in Table 1. ARCore (green) occasionally failed when the speed of the car increased or variations in the light abruptly occurred. In T265 (blue), if the car stopped temporarily due to a stop signal or was driving too fast, the VIO algorithm diverged and failed to estimate the location. ZED2 (magenta) accumulated rotational drift error over time, resulting in inaccurate motion estimation results. While the four VIO systems performed relatively well in the previous walking sequences, this was not the case in the more challenging vehicular test, which was not officially supported by any of the tested VIO devices. Only ARKit was able to produce stable motion-tracking results in the vehicular test.

**Figure 9:**
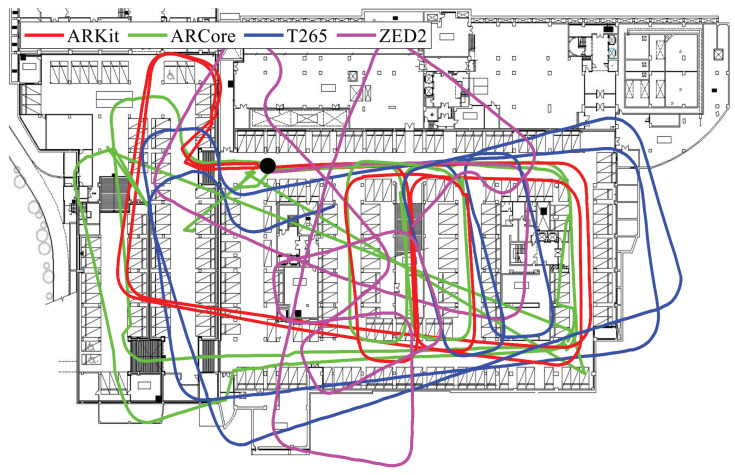
Example paths in the underground parking lot overlaid on the floor plan to evaluate the consistency and accuracy. The trajectories of ARKit (red) overlapped significantly, but the paths of the other VIO devices suffered from a rotational drift, showing inaccurate and inconsistent positioning results.

We conducted an additional vehicular test in which the same trajectory was driven repeatedly in a dark underground parking lot with poor visual conditions, as shown in Figure 9. The total traveling distance was about 450 m, and we drove the car at a low speed, from 5 to 15 km/h. Although ARKit did not perfectly restore the actual movements in the parking lot, ARKit (red) showed overlapped and consistent motion estimation results, while the other VIO systems gradually diverged from the initially estimated loop. Since we performed the evaluation at a relatively low speed (10 km/h) compared to the previous vehicle test (60 km/h), the other VIO systems did not diverge or fail at all. Among the four VIO methods, the positioning results of ZED2 are the most deviated from the actual movements in the underground parking lot.

### 4.5. Ground-Truth Comparison with OptiTrack

**Figure 10:**
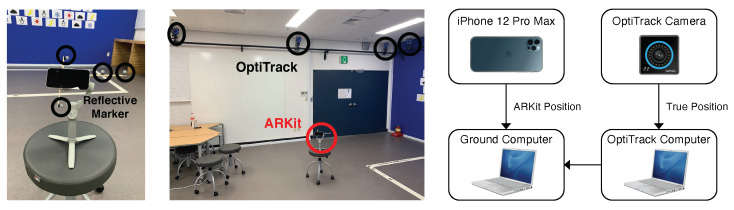
Experimental setup for the quantitative evaluation of ARKit. Four reflective markers were attached to the iPhone device (ARKit) to obtain the ground-truth moving trajectories from the OptiTrack motion capture systems. We acquired the true position of the VIO sensor platform in the 3D space and quantitatively evaluated the positioning accuracy of Apple ARKit.

We performed more comparison experiments with the actual moving trajectories (the ground truth) of an iPhone device (ARKit) from an OptiTrack motion capture system, as shown in Figure 10. We selected Apple ARKit in the iPhone 12 Pro Max as a VIO sensor because it showed the most accurate positioning performance among the commercial VIO sensors. We attached four reflective markers around the iPhone device to obtain the ground-truth moving trajectories in 3D space from OptiTrack motion capture systems.

**Figure 11:**
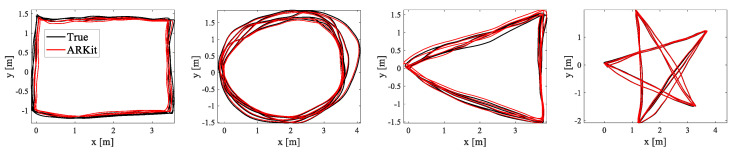
VIO sensor trajectories with ARKit (red) and the ground truth (black). The red and black lines represent the estimated and ground-truth trajectories from ARKit and the OptiTrack motion capture systems, respectively. The true trajectories (black) and the trajectories estimated with Apple ARKit (red) overlapped significantly.

We first defined rectangle-, circle-, triangle-, and star-shaped trajectories for the qualitative and quantitative evaluations and repeatedly moved along them for ‘multiple-loop’ trajectories, as shown in Figure 11. We collected the estimated (ARKit) and ground-truth (OptiTrack) trajectories in closed-loop sequences in which the starting and end points coincided. Figure 11 shows that the estimated trajectories (red) obtained from Apple ARKit were almost similar to the actual moving trajectories (black). In particular, due to the very small drift error in ARKit, the starting and end points were almost identical even after several turns of the multiple closed-loop trajectories. We measured the root mean squared error (RMSE) of the relative pose error (RPE) [37] and the final drift error (FDE) metric, and we present the quantitative evaluation results in Table 2. Both the qualitative and quantitative experimental results show that Apple ARKit was the most accurate among the four tested VIO sensors, and there was little difference when it was compared with the ground-truth trajectories.

## 5. Discussion

Overall, **Apple ARKit** demonstrated the most consistent, accurate, reliable, and stable motion-tracking results among the four VIO systems across both indoor and outdoor uses. ARKit performed well and robustly in various challenging real-world environments, such as environments with sudden camera movements, abrupt changes in illumination, and high-speed movements, with very rare cases in which tracking failure or motion jump occurred. ARKit achieved accurate and robust positioning performance in realistic use cases that were crowded with people and vehicles and were not only indoors, but also outdoors.

**Google ARCore** exhibited accurate and consistent motion-tracking performance next to ARKit. ARCore worked well for indoor sequences and the motion data collected by a walking person, but it diverged or the VIO algorithm deteriorated sharply when moving rapidly or in poor lighting conditions.

**Intel RealSense T265** showed good positioning performance that was just behind that of Google ARCore. T265’s operation of 6-DoF indoor motion tracking was not bad, but it had a problem with a scale inconsistency issue when estimating moving paths that were larger or smaller than the scale of the actual movements. In addition, T265’s motion tracking sometimes failed if the moving speed was too slow or fast.

The motion-tracking performance of **Stereolabs ZED 2** was the most inconsistent and inaccurate among the four VIO devices, both indoors and outdoors. As the 6-DoF motion tracking progressed, very severe rotational errors occurred, and these rotation errors accumulated over time, resulting in an incorrect path in which the starting and ending points were very different. In particular, ZED2 exhibited a tendency in which it could not correctly track a straight path when it was actually moving in a straight line outdoors, and the rotational drift error was more severe when moving fast.

We summarized the economic data and other important characteristics for each proprietary VIO sensor, as shown in Table 3. We collected the data in Table 3 based on the official website of each VIO sensor and the experimental results in our paper.

Since Apple ARKit and Google ARCore are 6-DoF motion-tracking algorithms that are designed for smartphone OSs (iOS, iPadOS, and Android), they operate only on specific mobile devices that support the corresponding OS. Although they have the advantage of being very accurate and stable, they lack compatibility and convenience in robot operating systems (ROSs) and Linux environments. On the other hand, T265 and ZED2 have excellent compatibility and convenience in ROSs and Linux environments that are used for various embedded computers, but lack accuracy and stability compared to ARKit and ARCore.

## 6. Conclusions

We conducted a survey of the 6-DoF ego-motion-tracking performance of four proprietary off-the-shelf VIO sensors in challenging real-world indoor and outdoor environments. To the best of our knowledge, this is the first back-to-back comparison of ARKit, ARCore, T265, and ZED2, and it demonstrated that Apple ARKit performed well and robustly in most indoor and outdoor scenarios. Apple ARKit showed the most stable and high accuracy/consistency, and the relative pose error was about 0.02 m of drift error per second. Although ARKit and ARCore have the advantage of being very accurate and stable, they lack compatibility and convenience in robot operating systems (ROSs) and Linux environments. On the other hand, T265 and ZED2 have excellent compatibility and convenience in ROSs and Linux environments that are used for various embedded computers, but lack accuracy and stability compared to ARKit and ARCore. We hope that the results and discussion presented in this paper may help members of the research community in finding appropriate VIO sensors for their robotic systems and applications.

## Figures and Tables

**Table 1:** Evaluation results (FDE) of the four proprietary VIO sensors.

Experiment	ARKit	ARCore	T265	ZED 2	Length (m)
Indoor Corridor	0.79	**0.12**	1.88	1.44	145.21
Indoor Hallway	0.14	**0.09**	0.61	4.58	83.98
Indoor Stairs	**0.19**	3.98	1.49	4.76	114.13
Outdoor Campus	2.01	**0.07**	4.08	206.38	513.81
Parking Lot	**0.26**	1.14	9.01	10.85	446.26
Outdoor Roads	**2.68**	140.08	×	409.25	3051.61

**Table 2:** Quantitative evaluation results for Apple ARKit.

Experiment	Relative Pose Error (RPE) (m/s)	Final Drift Error (FDE) (m)	Length (m)
Rectangle	0.02	0.09	50.46
Circle	0.02	0.02	68.69
Triangle	0.04	0.26	58.94
Star	0.03	0.17	61.84

**Table 3:** Economic comparison of the four commercial VIO sensors.

VIO System	Update Rate	Price (USD)	Pose Accuracy	Compatibility	System Requirements
ARKit	∼ 60 Hz	$429 ∼	+++	+	iOS (A9 and up)
ARCore	∼ 60 Hz	$449 ∼	+++	+	Supported Devices ${}^{1}$
T265	200 Hz	$329	++	+++	Single-Board Computer ${}^{2}$
ZED2	100 Hz	$449	+	++	NVIDIA GPU ${}^{3}$

^1^https://developers.google.com/ar/devices, accessed on 19 October 2022. ^2^ Since T265 computes all motiontracking data on the device, the only hardware requirement is a USB connection that provides 1.5Wof power. ^3^ NVIDIA Jetson Nano, TX2, Xavier, etc.

## Supplementary Materials

The following supporting information can be downloaded at: https://www.mdpi.com/article/10.3390/s22249873/s1, Video S1: More details about the experiments.
